# Targeting cyclin-dependent kinase 9 by a novel inhibitor enhances radiosensitization and identifies Axl as a novel downstream target in esophageal adenocarcinoma

**DOI:** 10.18632/oncotarget.27095

**Published:** 2019-07-23

**Authors:** Omkara Lakshmi Veeranki, Zhimin Tong, Rashmi Dokey, Alicia Mejia, Jianhu Zhang, Yawei Qiao, Pankaj Kumar Singh, Riham Katkhuda, Barbara Mino, Ramesh Tailor, Jaime Rodriguez Canales, Roland Bassett, Jaffer Ajani, Ji Yuan Wu, Scott Kopetz, Mariela Blum, Wayne Hofstetter, Michael Tetzlaff, Sunil Krishnan, Steven H. Lin, Dipen Maru

**Affiliations:** ^1^ Department of Pathology, The University of Texas MD Anderson Cancer Center, Houston, Texas, USA; ^2^ Experimental Radiation Oncology, The University of Texas MD Anderson Cancer Center, Houston, Texas, USA; ^3^ Translational Molecular Pathology, The University of Texas MD Anderson Cancer Center, Houston, Texas, USA; ^4^ Radiation Physics, The University of Texas MD Anderson Cancer Center, Houston, Texas, USA; ^5^ Biostatistics, The University of Texas MD Anderson Cancer Center, Houston, Texas, USA; ^6^ Gastrointestinal Medical Oncology, The University of Texas MD Anderson Cancer Center, Houston, Texas, USA; ^7^ Thoracic and Cardiovascular Surgery, The University of Texas MD Anderson Cancer Center, Houston, Texas, USA; ^8^ Radiation Oncology, The University of Texas MD Anderson Cancer Center, Houston, Texas, USA

**Keywords:** CDK 9 Inhibitor, esophageal adenocarcinoma, radiation, Axl

## Abstract

Cyclin-dependent kinase 9 (CDK9) transcriptionally regulates several proteins and cellular pathways central to radiation induced tissue injury. We investigated a role of BAY1143572, a new highly specific CDK9 inhibitor, as a sensitizer to radiation in esophageal adenocarcinoma. *In vitro* synergy between the CDK9 inhibitor and radiation was evaluated by clonogenic assay. *In vivo* synergy between the CDK9 inhibitor and radiation was assessed in multiple xenograft models including a patient’s tumor derived xenograft (PDX). Reverse phase protein array (RPPA), western blotting, immunohistochemistry, and qPCR were utilized to identify and validate targets of the CDK9 inhibitor. The CDK9 inhibitor plus radiation significantly reduced growth of FLO-1, SKGT4, OE33, and radiation resistant OE33R xenografts and PDXs as compared to the cohorts treated with either single agent CDK9 inhibitor or radiation alone. RPPA identified Axl as a candidate target of CDK9 inhibition. Western blot and qPCR demonstrated reduced Axl mRNA (*p* = 0.02) and protein levels after treatment with CDK9 inhibitor with or without radiation in FLO-1 and SKGT4 cells. Axl protein expression in FLO-1 xenografts treated with combination of CDK9 inhibitor and radiation was significantly lower than the xenografts treated with radiation alone (*p* = 0.003). Clonogenic assay performed after overexpression of Axl in FLO-1 and SKGT4 cells enhanced radiosensitization by the CDK9 inhibitor, suggesting dependency of radiosensitization effects of the CDK9 inhibitor on Axl. In conclusion, these findings indicate that targeting CDK9 by BAY1143572 significantly enhances the effects of radiation and Axl is a novel downstream target of CDK9 in esophageal adenocarcinoma.

## INTRODUCTION

The incidence of adenocarcinoma of the esophagus and gastro esophageal junction has rapidly increased in the USA and other western countries over past 30 years [1, 2]. Majority of patients with esophageal adenocarcinoma present with loco-regional (stage II–III) disease. Esophagogastrectomy had been the standard of care for these patients for many years. In the last decade, advent of preoperative chemoradiation in neoadjuvant setting has improved patients’ survival and likelihood of complete surgical resection [3, 4]. In spite of such aggressive therapeutic approach, 5-year survival for these patients is 20–30% [5, 6]; primarily because of development of chemoradiation resistance and inability of chemoradiation to kill all tumor cells to achieve complete pathologic response. Molecular targeted therapy is yet to show efficacy in enhancing chemoradiation efficacy in neoadjuvant setting in esophageal adenocarcinoma. A phase III study (RTOG 1010) evaluating role of Trastuzumab to enhance response to chemoradiotherapy and surgery is ongoing, although positive findings will likely benefit only up to 15–20% of patients as frequency of HER2-neu overexpression is observed in <20% of esophageal adenocarcinoma [7]. Therapies targeting EGFR and VEGF have failed to show substantial improvement in patient outcome [8, 9]. Major limiting factor for successful implementation of molecular targeted therapy is low frequency and heterogeneity of alterations in targets like Her2-neu amplification/overexpression (15%), EGFR amplification (20%), EGFR activating mutations (0–12%) and c-MET amplification (2–10%) [7, 9–11]. The unmet need to improve efficacy of radiation in esophageal adenocarcinoma has been recognized as a strategic priority by the NCI Gastrointestinal Steering Committee [12]. Moreover, enhancing radiosenstization by a targeted agent has an advantage of reducing the required dose and toxicity of radiotherapy to the vital organs like heart and lungs.

Cyclin dependent kinase (CDK) 9 is a promising target to enhance radiosensitization [13–16]. Previously, we showed overexpression of CDK9 in esophageal adenocarcinoma cells compared to matched normal esophageal epithelial cells and Barrett’s esophagus [16]. BAY1143572 (Atuveciclib), a novel, first-in-class CDK9 specific inhibitor more potently inhibits CDK9 (PTEFb) activity because it’s IC50 is 50 fold lower than the IC50 of other CDKs [17–19]. BAY1143572 has high specificity for CDK9 at the nanomolar level without any off-target toxicity, unlike other CDK9 inhibitors [17, 20]. A recent study showed strong potential of BAY1143572 as a novel treatment for adult T-cell leukemia/lymphoma [19]. BAY1143572 induces its anti-tumorigenic effects in adult T-cell leukemia/lymphoma by inhibiting pSer2 and pSer7 RNA Pol II, MYC, and MCL-1. Our studies [16] (unpublished data) indicate that CDK9/p-TEFb inhibition is the dominant mechanism of action for three CDK inhibitors: Flavopiridol [21, 22], CAN 508 and BAY1143572, in esophageal adenocarcinoma. In the present study, we show for the first time that inhibition of CDK9 potently enhances radiation sensitivity in various preclinical models of esophageal adenocarcinoma. We further demonstrate that Axl, a tyrosine kinase critical in determining radiation sensitivity in solid tumors, is a novel target of CDK9 inhibitor with and without radiation in esophageal adenocarcinoma.

## RESULTS

### CDK9 inhibitor enhances sensitization of esophageal adenocarcinoma to radiation *in vitro*

Previously, we showed that CDK9 inhibitors exert dose-dependent anti-proliferative effects against 6 esophageal adenocarcinoma cell lines [16]. Radioresistant OE33R cells were established by exposing radiosensitive OE33 cells to weekly doses of 2 Gy radiation and radiation resistance was achieved after 45 fractions of 2 Gy radiation. In the present study, MTS assay confirmed dose-dependent anti-proliferative effects of BAY1143572 in radiosensitive OE33 and radioresistant OE33R cells (Supplementary Figures 1 and 2A, 2B).

**Figure 1:**
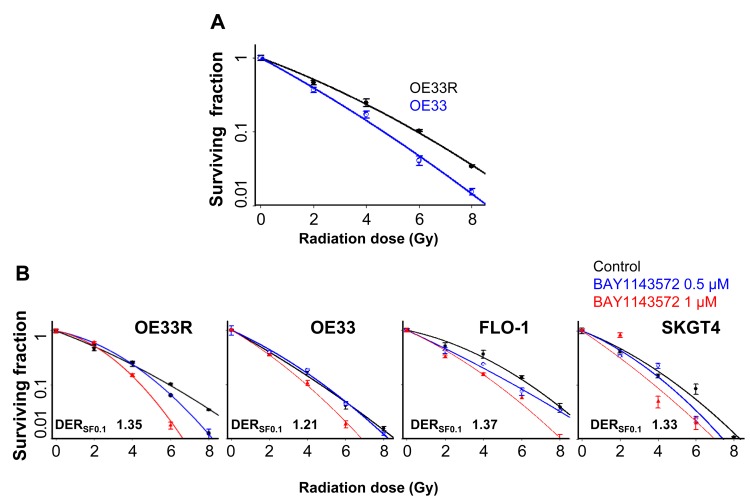
CDK9 inhibitor has potent radiosensitization efficacy in esophageal adenocarcinoma cells. (**A**) Clonogenic survival assay confirming radioresistance in OE33 radio resistant cells compared to radionaïve OE33 cells. (**B**) Clonogenic survival assay for FLO-1, SKGT4 and radionaïve and radio resistant OE33 cells, treated with (0.5, 1 μM) BAY1143572 for 24 hours and irradiated (2, 4, 6, 8 Gy) at 5 hours post inhibitor treatment demonstrated synergy between CDK9 inhibitors and radiation in esophageal adenocarcinoma cell lines. Dose enhancement ratio at survival fraction (DER_SF0.1_) of 10% was calculated by (radiation dose needed to kill 90% without drug)/(radiation dose needed to kill 90% with drug). The radiation dose was calculated from the linear quadratic model based on the survival fraction at each dose. A DER _SF0.1_ value of ≥1.1 indicates synergy. Data are derived from three independent experiments conducted in triplicates.

The clonogenic survival assay demonstrated significantly higher survival fractions among the OE33R cells compared to OE33 at 4, 6 or 8 Gy (Figure 1A) confirming radiation resistance in OE33R cells. However, treatment with BAY1143572 had the highest degree of sensitization at a survival fraction of 10% (DER_SF0.1_) in FLO-1 cells (DER_SF0.1_, 1.37), followed by radioresistant OE33R cells (DER_SF0.1_, 1.35), SKGT4 cells (DER_SF0.1_, 1.33) and radiosensitive OE33 cells (DER_SF0.1_, 1.21) (Figure 1B).

### CDK9 inhibitor sensitizes esophageal adenocarcinoma xenografts to radiation

**Figure 2:**
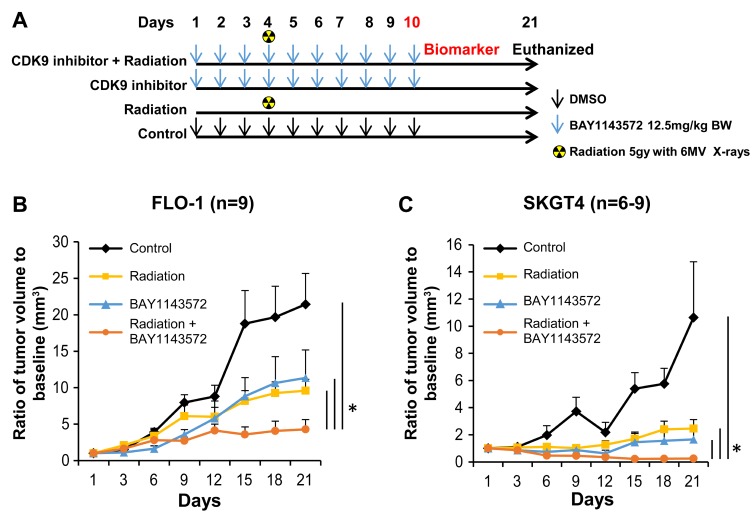
CDK9 inhibitor enhances sensitization of radiation in esophageal adenocarcinoma xenografts. Targeting CDK9 by BAY1143572 demonstrated sensitization of two esophageal adenocarcinoma xenografts to radiation (**A**) Dosing regimen of four different treatment cohorts. (**B**) BAY1143572 enhances radiation induced tumor regression in FLO-1 and (**C**) SKGT4 xenografts. Nude mice were subcutaneously injected with 5 × 10^6^ FLO-1 cells or SKGT4 to generate ectopic xenografts. Tumor volume was normalized to baseline tumor volume at day 1 of treatment. Ratio of tumor volume to baseline was measured to assess tumor growth over 21 days since start of treatment. Data are the mean percentages of tumor growth ± SE. ^*^ *p* < 0.05 compared between control and other cohorts.

**Figure 3:**
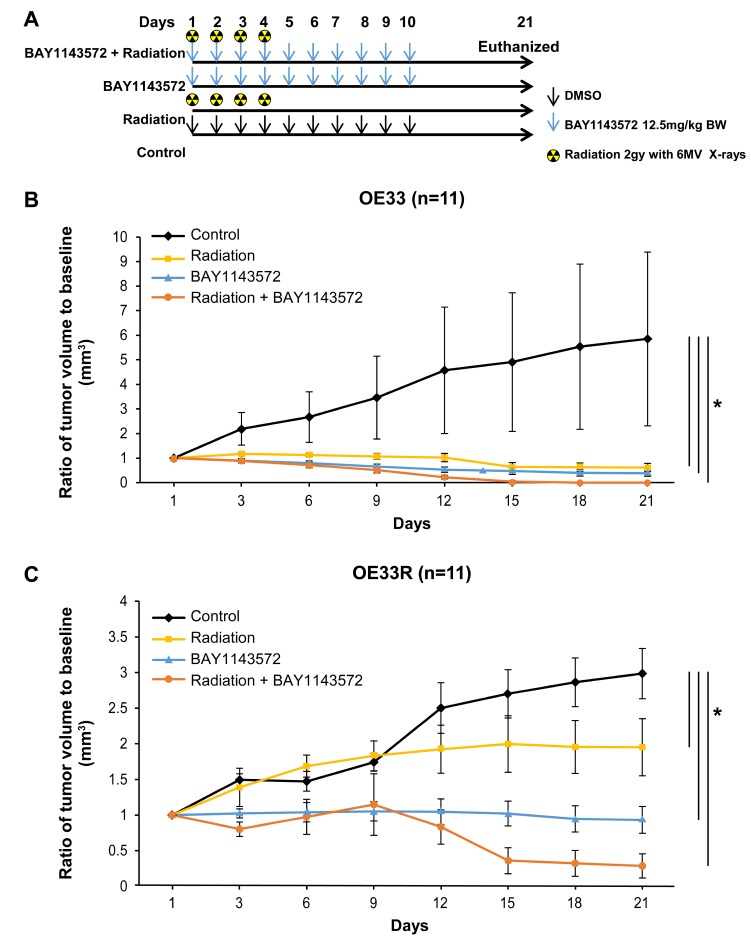
CDK9 inhibitor enhances sensitization of fractionated radiation in OE33 and OE33R xenografts. Targeting CDK9 by BAY1143572 in radiosensitive OE33 and radioresistant OE33R xenografts demonstrated sensitization of both xenografts to radiation (**A**) Dosing regimen of four different treatment cohorts. (**B**) BAY1143572 enhances radiation induced tumor regression in OE33 and (**C**) OE33R xenografts. Nude mice were subcutaneously injected with 5 × 10^6^ OE33 or OE33R cells to generate ectopic xenografts. Tumor volume was normalized to baseline tumor volume at day 1 of treatment. Ratio of tumor volume to baseline was measured to assess tumor growth over 21 days since start of treatment. Data are the mean percentages of tumor growth ± SE. ^*^ *p* < 0.05 compared between control and other cohorts.

The treatment cohorts and dosing regimen are shown in Figures 2A and 3A. At day 21, the mean volumes of FLO-1 xenografts treated with 12.5 mg/kg BAY1143572 only or 12.5 mg/kg plus radiation were 67% and 48% smaller than that of the control arm (Figure 2B). The mean volume of the CDK9 inhibitor -plus-radiation–treated xenografts was 63% smaller than that of xenografts treated with radiation only (*p* = 0.01) and 42% smaller than that of xenografts treated with the CDK9 inhibitor only (*p* = 0.05).

Slow growing SKGT4 xenografts (Figure 2C) responded better to the CDK9 inhibitor plus radiation than FLO-1 xenografts. At day 21, the mean volumes of xenografts treated with 12.5 mg/kg CDK 9 inhibitor only or 12.5 mg/kg CDK9 inhibitor plus radiation were 84% (*p* = 0.03) and 98% (*p* = 0.02) smaller than that of the control arm (Figure 2C). The mean volume of the CDK9 inhibitor -plus-radiation–treated xenografts was 90% smaller than that of xenografts treated with radiation only (*p* = 0.005) and 85% smaller than that of xenografts treated with the CDK9 inhibitor only (*p* = 0.06). The mice showed no significant signs of toxicity throughout the treatment period [body weights at D21 – control (27.07 ± 0.89), radiation (29.10 ± 0.60), CDK 9 inhibitor (26.50 ± 0.78) and combination (27.12 ± 0.69)].

Growth inhibition of radiation-naïve OE33 xenografts was evident from the onset of treatment with CDK9 inhibitor or radiation. At day 21, the mean volumes of OE33 xenografts treated with 12.5 mg/kg CDK9 inhibitor or 12.5 mg/kg CDK9 inhibitor plus a total of 8 Gy of radiation were 94% (*p* = 0.005) and 100% (*p* = 0.005) smaller, respectively, than that of control xenografts (Figure 3B). At day 21, the mean volumes of OE33R xenografts treated with 12.5 mg/kg CDK9 inhibitor or 12.5 mg/kg CDK9 inhibitor plus 8GY of radiation were 68.8% and 90.5% (*p* < 0.001) smaller, respectively, that that of control xenografts (Figure 3C). The mean volume of the CDK9 inhibitor-plus-radiation–treated xenografts was 85% smaller than that of xenografts treated with radiation only and 69% smaller than that of xenografts treated with the CDK9 inhibitor only (*p* = 0.01) in OE33R xenografts. The mean volume of the CDK9 inhibitor-plus-radiation–treated xenografts was 100% smaller than that of xenografts treated with radiation only or CDK9 inhibitor only in OE33 cells.

The mice showed no significant signs of toxicity throughout the treatment period [body weights at D21 – control (25.12 ± 0.53), radiation (27.23 ± 0.98), CDK9 inhibitor (23.50 ± 0.42) and combination (26.03 ± 0.74)].

Tumor cell necrosis in xenografts can be due to cytotoxic effects of the treating agents or due to tumor cells outgrowing the vascular supply. It is not possible to differentiate ischemic necrosis or drug induced cytotoxicity in cell line derived xenografts due to lack of stromal elements. To normalize the tumor cell necrosis to the rate of tumor growth, we assessed percentage of tumor necrosis divided by the tumor growth rate (mm/day) to deduce necrosis induced by the treatment. As shown in Table 1, CDK9 inhibitor plus radiation-induced tumor regression was associated with necrosis (*p* = 0.037 compared to radiation alone).

**Table 1:** Mean tumor necrosis/rate of growth (mm/day) in FLO-1 xenografts

FLO-1 xenografts	Mean tumor necrosis/rate of growth (mm/day)	*p*-value
Control	0.10	
Radiation	0.06	0.23^*^
BAY1143572	0.50	0.072^**^, 0.055^****^
Radiation _+_ BAY1143572	0.40	0.054^***^, 0.037^*****^, 0.38^******^

^*^= Radiation vs. control.

^**^= BAY1143572 vs. control.

^***^= Radiation + BAY1143572 vs. control.

^****^= BAY1143572 vs. radiation.

^*****^= Radiation + BAY1143572 vs. Radiation.

^******^= Radiation + BAY1143572 vs. CDK9 inhibitor.

Comparison of mean tumor necrosis/rate of growth (mm/day) across different treatment cohorts.

### CDK9 inhibitor radiosensitizes PDXs from a treatment refractory esophageal adenocarcinoma

**Figure 4:**
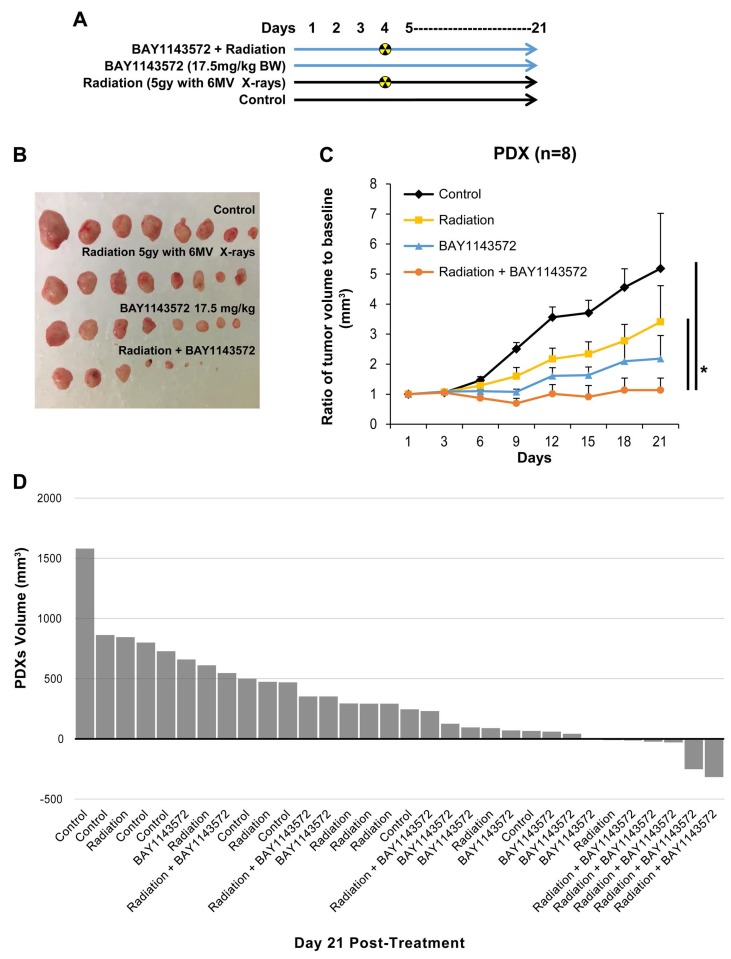
CDK9 inhibitor enhances sensitization of radiation in esophageal adenocarcinoma PDXs. Pharmaceutical inhibition of CDK9 by BAY1143572 at 17.5 mg/kg body weight in esophageal adenocarcinoma PDX sensitized tumors to radiation (5 Gy with a 6MV X-rays on day 4 of treatment). (**A**) Dosing regimen of four different treatment cohorts. (**B**) PDX tumor volume comparison by macroscopic examination of excised tumors on day 21 in different treatment cohorts. (**C**) Demonstrating results of efficacy of BAY1143572 with and without radiation therapy in PDX. Tumor volume was normalized to baseline tumor volume at day 1 of treatment. Ratio of tumor volume to baseline was measured to assess tumor growth over 21 days since start of treatment. Figure represents ratio of tumor growth rate in PDX. Data are the mean percentages of tumor growth ± SE. ^*^ *p* < 0.05 compared between control and other cohorts. (**D**) Waterfall plot of tumor volume at day 21 post BAY1143572 treatment shows five of eight tumors from combination group to be smaller than baseline.

PDXs were generated from a 45-year-old Caucasian man with HER2/neu negative stage IV esophageal adenocarcinoma with widespread systemic metastases refractory to therapy (Supplementary Figure 3). Treatment with 17.5 mg/kg the CDK9 inhibitor for 20 days plus 5 Gy of radiation once on day 4 (Figure 4A) reduced PDX tumor volumes by 67% compared with radiation alone (*p* = 0.016) and by 48% compared with the CDK9 inhibitor alone (*p* = 0.062) (Figure 4B and 4C). As seen in waterfall plot (Figure 4D), 5 of 8 xenografts were smaller after treatment with the combination than at baseline, whereas 1 of 8 xenografts were smaller after treatment with either treatment alone. No significant toxicity was observed in mice treated with CDK9 inhibitor.

PDXs have patient tumor stroma in contrast to cell line–derived xenografts; therefore, it was possible to assess treatment-induced stromal fibrosis in PDXs. The median percent tumor fibrosis for control, CDK9 inhibitor-, radiation-, and combination-treated cells were 15, 10, 20, and 30, respectively (Table 2). Tumor growth inhibition was associated with CDK9 inhibitor-mediated fibrosis, which was significantly elevated in the combination group compared with the control (*p* = 0.002), radiation (*p* = 0.002), and CDK9 inhibitor (*p* = 0.009) groups.

**Table 2:** Median percent tumor fibrosis by BAY1143572 in PDX at day 21

Patient derived xenografts	Median % tumor fibrosis [Range]	*p*-value
Control	15 [10–30]	
Radiation	10 [5–20]	0.26^*^, 0.002^****^
BAY1143572	20 [10–30]	0.17^**^, 0.009^*****^
Radiation _+_ BAY1143572	30 [20–35]	0.002^***^

^*^ = Control vs Radiation.

^**^ = Control vs BAY1143572.

^***^ = Control vs Radiation _+_ BAY1143572.

^****^ = Radiation vs Radiation _+_ BAY1143572.

^*****^ = BAY1143572 vs Radiation _+_ BAY1143572.

Results indicate that combination group showed significantly higher tumor fibrosis compared to all other cohorts. The *p*-values were calculated using the Student *t* test.

### Axl as a target of CDK9

**Figure 5:**
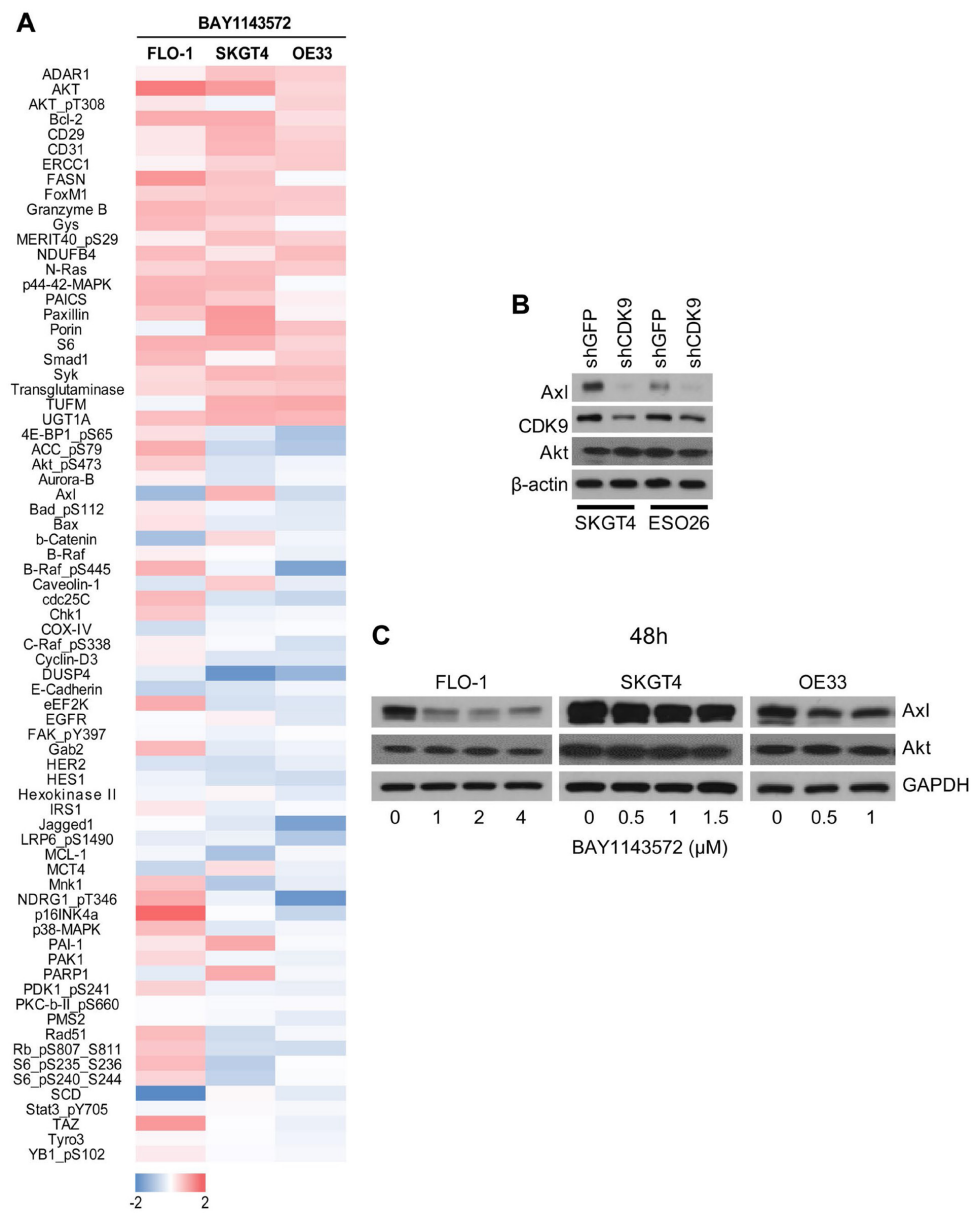
Effect of the CDK9 inhibitor on protein expression reverse phase array (RPPA) and identification of Axl as a novel downstream target of CDK9. (**A**) RPPA was performed on FLO-1, SKGT4 and OE33 cells treated with or without BAY1143572. RPPA marker expression is denoted by red indicating overexpression compared to control cell lysates and blue indicating downregulation compared to control lysates. (**B**) Western blot images of downregulated Axl in shCDK9 clones of two esophageal adenocarcinoma cells: SKGT4, and ESO26. Akt and β-actin were used as loading control. (**C**) Western blot images of BAY1143572 treated for 48 hours (48 h) in FLO-1, SKGT4 and OE33 cells show dose dependent decrease in total Axl protein compared to control lysates by western blot. Akt and GAPDH were used as loading control.

RPPA analysis showed that, compared with the control cells, FLO-1, SKGT4, and OE33 cells treated with 1 μM CDK9 inhibitor for 48 hours had lower expression of oncoproteins such as MCL-1, mTOR, c-MET, E-cadherin, Axl, PMS2, and HES1 but higher expression of Brd4, FOXM1, and MERIT40 (Figure 5A and Supplementary Table 1). As MCL-1, mTOR, and c-MET are known CDK9 targets, we investigated Axl as a novel downstream target of CDK9 *in vitro* and in xenograft samples. Owing to technical challenges in obtaining reasonable downregulation of CDK9 by shCDK9 in FLO-1 and OE33 cells, we generated shCDK9-mediated genetic downregulation in SKGT4 and ESO26 cells. Genetic downregulation of CDK9 in both SKGT4 and ESO26 cells significantly lowered Axl protein expression compared to their control counterparts (Figure 5B), indicating that Axl downregulation is specific to CDK9 inhibition and that Axl is a downstream target of CDK9. Similarly, FLO-1, SKGT4 and OE33 cells treated with BAY1143572 (Figure 5C) had suppressed Axl protein levels compared to control cells, suggesting Axl may be regulated by CDK9 or CDK9 dependent pathways. Akt was used as a control in addition to housekeeping controls β-actin and GAPDH to confirm Axl downregulation by CDK9 inhibition.

**Figure 6:**
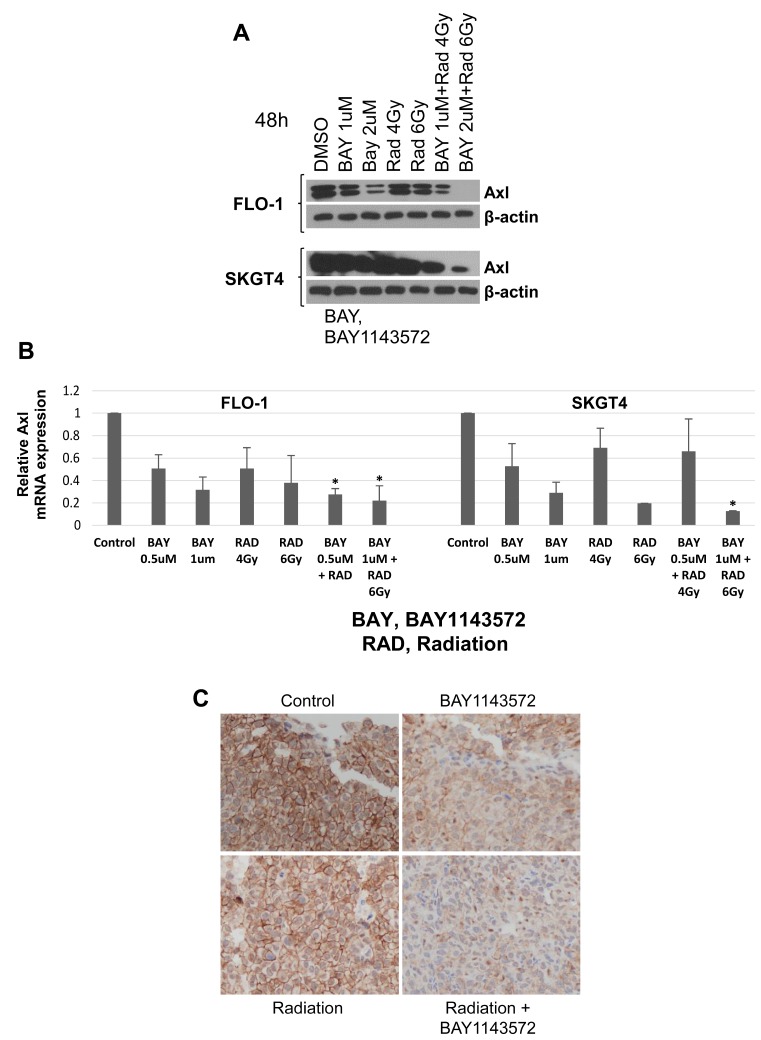
Axl is a novel downstream target of CDK9. (**A**) BAY, BAY1143572 showed synergistic decrease in total Axl protein when treated with 6 Gy radiation in FLO-1 and SKGT4 cells for 48h. β-actin was used as loading control. (**B**) qRT-PCR shows decreased Axl mRNA by BAY1143572 and radiation in two esophageal adenocarcinoma cell lines: FLO-1, and SKGT4. Parental cells treated with DMSO were used as controls in each case. Axl mRNA was normalized to GAPDH mRNA expression in this analysis. Combination groups is not synergistic to individual treatments. The *p*-values were calculated using the Student *t* test; ^*^T/C, *p*-value ≤ 0.05. (**C**) Immunohistochemistry staining for Axl at 100x magnification in FLO-1 xenografts treated with BAY1143572 with or without radiation.

Inhibition of CDK9 has shown downregulation of a large number of short-lived anti-apoptotic proteins such as MCL-1, thus time dependent Axl expression by CDK9 inhibition was determined at earlier time points (8 and 24 hours) at the protein and mRNA level (Supplementary Figure 4A and 4B). There was no change in Axl protein and mRNA (*p* > 0.05), 8 hours after treatment with BAY1143572 (0.5 and 1μM), as compared to control in FLO-1 and SKGT4 cells. However, at 24 hours, 1μM BAY1143572 treated FLO-1 and SKGT4 showed slight downregulation of Axl protein as compared to the control. BAY1143572 (0.5 and 1μM) decreased Axl RNA in FLO-1 and SKGT4 cells compared to control cells (*p* < 0.05) at 24 hours after treatment. Axl was further downregulated 48 hours after treatment with CDK9 inhibitor (Figure 6A), confirming the RPPA and genetic CDK9 downregulation results. MCL-1 was downregulated as early as 4 hours after the CDK9 inhibitor treatment (Supplementary Figure 5).

Radiation alone reduced Axl protein expression at 48 hours. CDK9 inhibitor in combination with radiation further downregulated Axl and MCL-1 in FLO-1, and SKGT4 cells.

qRT-PCR demonstrated that Axl RNA was decreased in FLO-1, and SKGT4 cells irradiated with 4 or 6 Gy for 24 hours. Cells treated with 1 μM CDK9 inhibitor alone or in combination with radiation had lower Axl RNA levels compared to control cells (*p* < 0.05, and CDK9 inhibitor plus radiation modestly decreased Axl RNA more than either treatment alone did (Figure 6B).

### CDK9 inhibitor enhances reduction of Axl protein expression by radiation in FLO-1 xenografts

The mean (range) H-scores of Axl staining on xenografts treated with radiation or the CDK9 inhibitor alone were significantly lower than that of the control xenografts (*p* = 0.02 and *p* = 0.007, Figure 6C or Table 3). The mean H-scores of Axl staining on xenografts treated with combination of the CDK9 inhibitor with radiation were significantly lower than xenografts treated with radiation alone (*p* = 0.003 Figure 6C or Table 3). Untreated control SKGT4 tumors and PDXs had very low to undetectable Axl expression, precluding analysis of effects of different treatments on Axl expression in these xenografts.

**Table 3:** Quantitative immunohistochemistry analysis of Axl membranous staining in tumor cells of the FLO-1 xenografts

FLO-1 xenografts	Mean % of tumor cells with 0 staining	Mean % of tumor cells with 1 staining	Mean % of tumor cells with 2 staining	Mean % of tumor cells with 3 staining	H-score, Mean (Range)	p-value
Control	5	5	80	10	120 (80-195)	
Radiation	65	25	10	0	65 (10-90)	0.02^*^
BAY1143572	**75**	**25**	**5**	**0**	20 (5-65)	0.007^**^ **,** 0.06^***^
Radiation _+_ BAY1143572	**80**	**20**	**0**	**0**	20 (5-60)	0.003^****^ **,** 0.48^*****^

^*^= Radiation vs. control, ^**^= BAY1143572 vs. control, ^***^= BAY1143572 vs. radiation, ^****^= Radiation + BAY1143572 vs. Radiation, ^*****^= Radiation + BAY1143572 vs. BAY1143572.

### CDK9 inhibitor enhances radiosensitization of esophageal adenocarcinoma cells with overexpressed Axl

**Figure 7:**
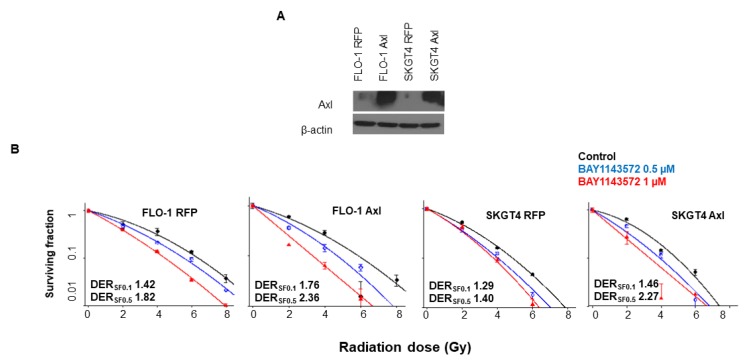
Overexpression of Axl enhanced radiosensitization by CDK9 inhibitors in esophageal adenocarcinoma cells. (**A**) Western blot images of overexpressed Axl in two esophageal adenocarcinoma cells: FLO-1, and SKGT4. β-actin was used as loading control. (**B**) Clonogenic survival assay in FLO-1 and SKGT4 cells with or without Axl overexpression treated with (0.5, 1 μM) BAY1143572 for 24 hours and irradiated (2, 4, 6, 8 Gy) at 5 hours post inhibitor treatment demonstrated enhanced synergy by combination treatment in overexpressed Axl esophageal adenocarcinoma cells. Dose enhancement ratio at survival fraction (DER_SF0.1_) of 10% was calculated by (radiation dose needed to kill 90% without drug)/(radiation dose needed to kill 90% with drug). The radiation dose was calculated from the linear quadratic model based on the survival fraction at each dose. A DER _SF0.1_ value of ≥1.1 indicates synergy. Data are derived from three independent experiments conducted in triplicates.

To evaluate, whether suppression of Axl by CDK9 inhibition is associated with enhanced sensitization to radiation, we stably overexpressed Axl in two esophageal adenocarcinoma cells FLO-1 and SKGT4 (Figure 7A). The clonogenic survival assay demonstrated enhanced sensitization to radiation by BAY1143572 in esophageal adenocarcinoma cells with stable overexpression of Axl (FLO-1 Axl and SKGT4 Axl) compared to negative control cells (FLO-1 RFP and SKGT4 RFP) OE33 at 4, 6 or 8 Gy (Figure 7B). These results indicate that cells with overexpressed Axl are relatively more sensitive to combination therapy compared to negative control cells. Treatment with BAY1143572 had the higher degree of sensitization at a survival fraction of 10% (DER_SF0.1_) and 50% (DER_SF0.5_) in FLO-1 Axl cells (DER_SF0.1_, 1.76; DER_SF0.5_, 2.36) compared to its control FLO-1 RFP cells (DER_SF0.1_, 1.42; DER_SF0.5_, 1.82). Similarly, BAY1143572 exhibited higher degree of sensitization in SKGT4 Axl cells (DER_SF0.1_, 1.46; DER_SF0.5_, 2.27) compared to its control SKGT4 RFP (DER_SF0.1_, 1.29; DER_SF0.5_, 1.40) (Figure 7B).

## DISCUSSION

This study provides strong evidence of efficacy of a novel CDK9 inhibitor in preclinical models of esophageal adenocarcinoma, supporting a role of targeted inhibition of CDK9 as sensitizer to the radiation in a clinical trial of esophageal adenocarcinoma.

Prior studies demonstrated a better pharmaceutical profile with high aqueous solubility of 479 mg/L of BAY1143572 as well as decreased efflux ratio and far better tolerability *in vivo* compared to other CDK9 inhibitors. These pharmacologic properties distinguish BAY1143572 from other CDK9 inhibitors in terms of potency, drug safety, and tolerability and enabled BAY1143572 to be the first highly selective PTEFb/CDK9 inhibitor to enter phase 1 clinical trials (NCT01938638 and NCT02345382) [18].

Clonogenic assay showing ≥1.1 DER_SF0.1_ in all but one tested esophageal adenocarcinoma cell lines suggest synergy between CDK9 inhibition and radiation in esophageal adenocarcinoma, despite possible heterogeneity across the cell lines. The additive radiation enhancing effect of CDK9 inhibitor was found to be greater at higher radiation doses, indicating a low likelihood of plateauing effects of radiation-mediated tumor cell damage.

Treatment with the CDK9 inhibitor plus radiation potently retarded the rapid growth of radiation sensitive FLO-1, OE33 xenografts, radiation resistant OE33R xenografts and slowly growing SKGT4 xenografts suggesting that the combination has potent anti-tumorigenic effects *in vivo* against esophageal adenocarcinoma. Xenografts treated with the CDK9 inhibitor and radiation shrunk to below baseline volumes, whereas those treated with radiation or a CDK9 inhibitor alone had retarded growth but did not shrink below baseline volumes, suggesting that the combination had higher efficacy than either treatment alone did. That the combination had higher efficacy is also supported by the high degree of tumor necrosis and stromal fibrosis in the xenografts after pretreatment with CDK9 inhibitor followed by radiation.

Although the establishment of PDX esophageal adenocarcinoma models was first described in 1981 [23, 24], very few targeted agents have been studied for anticancer efficacy [25, 26]. In the present study, PDXs were generated from an advanced-stage chemo-refractory subcutaneous tumor nodule with high genetic instability and were ectopically implanted, mimicking subcutaneous tumors in patients. These refractory PDXs responded to radiation, albeit to a lesser extent than to CDK9 inhibitors alone or in combination with radiation, suggesting that radiation could have achieved limited local control of the subcutaneous metastases in the patient. The ability of continuous single-agent BAY1143572 to suppress tumor regrowth after radiation indicates that this CDK9 inhibitor can maintain the anti-tumorigenic effects of radiation.

We found that, unlike previous generations of CDK9 inhibitors, BAY1143572 had no noticeable systemic toxicities. Gross inspections of the gastrointestinal tract and H&E staining of the liver, heart, and kidneys in all xenograft models revealed no or low toxicity. Regardless, studying the effects of this CDK9 inhibitor on radiation-induced esophagitis and toxicity to the peri-esophageal organs is warranted. Such effects will need to be evaluated in orthotopic xenograft mouse models, as subcutaneous models are not ideal for the assessment of local toxicities associated with multimodal therapies.

Importantly, our findings demonstrate that Axl is a new downstream target of CDK9 inhibition. Both the genetic downregulation and the pharmaceutical inhibition of CDK9 downregulated Axl, confirming the on-target effects of CDK9 on Axl. More pronounced effects of the CDK9 inhibitor on Axl levels during late post-treatment phase suggest that CDK9 inhibitor may be regulating Axl by indirect mechanism, where it would either regulate binding of a transcription regulator (HIF-1alpha [27, 28], etc.) to Axl or modifies another protein that is a regulator of Axl. Clonogenic assay in esophageal adenocarcinoma cells stably overexpressing Axl showed greater DER_SF0.1_ compared to their negative control suggesting increased sensitivity to combination therapy in Axl overexpressed cells. Thus downregulation of Axl by CDK9 inhibition may be one of the mechanisms by which CDK9 inhibition radiosensitizes esophageal adenocarcinoma cells.

Axl, a TAM (Tyro3/Axl/Mer) family receptor tyrosine kinase (RTK), was recently shown to be highly expressed in radio-resistant tumors, and Axl inhibition in combination with radiotherapy or other anticancer therapies elicited anti-tumor responses [29]. Given Axl’s roles in oncogenesis and therapy resistance, multiple agents targeting Axl are in development [30–34]. Several Axl inhibitors and monoclonal antibodies target the extracellular domains, kinase domains, or ligands of Axl [30, 33, 35, 36]; however, the inherent challenges these types of inhibitors pose; such as inability to recognize tumor cells with mutant ligands may be mitigated by CDK9’s transcriptional regulation of Axl, thus improving the impact of targeting Axl-mediated resistance to therapy. Additionally CDK9 inhibition has been shown to reactivate epigenetically silenced genes [37] which may potentially be involved in modulating Axl protein levels. A mechanistic study to elucidate the role of CDK9 in targeting Axl is warranted to identify regulation of transcription factors binding to Axl promoter by CDK9. However, due to multiple transcription initiation sites, GC rich promoter region, methylation within and around specific proteins (Sp) 1 and 3 binding sites, it is challenging to study transcription regulation of Axl [38, 39]. This will require testing transcription regulation of Axl promoter by more than one method so that majority of the promoter region is covered and quantitative data is generated that can be comparable across different treatment cohorts.

In summary, general failure of targeting CDK9 with pan-CDK inhibitors in clinics suggests that improved selectivity to CDK9 is the key to a successful development of CDK9 inhibitors as viable therapeutic agents. Our preclinical data suggest that CDK9 inhibition, particularly by BAY1143572, is a potential strategy for radio-sensitizing esophageal adenocarcinoma and support the investigation of BAY1143572 as an adjunct to radiation in clinical trials. Our findings also indicate that the interplay between CDK9 and Axl should be investigated as a novel mechanism of CDK9 inhibitor mediated radiosensitization in esophageal adenocarcinoma.

## MATERIALS AND METHODS

All mice experiments were conducted as per the institutional guidelines. De-identified patient samples were obtained with informed consent and xenografted as per institutional IRB and IACUC approved protocols (LAB-04-0979 and IACUC-00001501-RN01).

### Cell lines, cell culture, and CDK9 inhibitor

BAY1143572 was purchased from Active Biochemical (Wan Chai, Hong Kong). Human esophageal adenocarcinoma cell lines, FLO1, OE33, and SKGT4 were purchased from Sigma Aldrich or ATCC. Radiation resistant esophageal adenocarcinoma cells (OE33R) were provided by our institutional Center for Radiation Oncology Research. Complete cell line information is provided in the Supplementary Materials and Methods.

### Cell lines and tumor irradiation

Cells were irradiated with doses of up to 6 Gy using a JL Shepherd Mark I-68A ^137^Cs irradiator. The xenograft bearing hind legs of mice were irradiated using a 6-MV photon beam of a Varian 2300CD Linear Accelerator.

### Clonogenic survival assay

Esophageal adenocarcinoma cells were seeded on 6-well plates (in duplicates) at densities of 100-2000 cells/well. Sixteen hours after plating, the medium was changed, and the cells were treated with either vehicle (dimethyl sulfoxide [DMSO]) or CDK9 inhibitor. Five hours after treatment, the cells were irradiated with 0, 2, 4, 6, or 8 Gy. Twenty-four hours after radiation treatment, the medium was changed, and the cells were maintained in the normal culture conditions. Between days 12 and 20, the medium was removed, and cell colonies were stained with crystal violet (0.1% in 20% methanol) (Sigma-Aldrich) [40]. Colonies were assessed visually, and colonies containing >50 normal-appearing cells were manually counted. The surviving fraction was calculated using SigmaPlot 10.0 (CA, USA). The radiation dose enhancement ratio at the surviving fraction 0.1 (DER_SF0.1_) was calculated using the equation; DER_SF0.1_= Dose (radiation + control) /Dose (radiation+CDK9 inhibitor) at SF0.1. Synergy between a CDK 9 inhibitor and radiation against esophageal adenocarcinoma cells was defined as DER_SF0.1_ higher than 1.1, as described previously [41]**.**

### Reverse phase protein array (RPPA)

Cells were treated with 1 μM CDK9 inhibitor for 30 hours. Cell lysates were analyzed for protein expression by RPPA at our institutional Functional Proteomics Core Facility [41, 42]. Cluster 3.0 (http://bonsai.hgc.jp/~mdehoon/software/cluster/software.htm) with Pearson correlation and a center metric was used to hierarchically cluster data. The resulting heatmap was visualized with Treeview (http://www.eisenlab.org/eisen/) and presented in a high-resolution bmp format.

### Efficacy of CDK9 inhibitor with and without radiation against esophageal adenocarcinoma xenografts

Four-week-old female athymic nude mice were used for xenograft studies. All experiments involving mice were conducted according to an animal experimental protocol approved by Institutional Animal Care and Use Committee. To assess the radiosensitizing effects of CDK9 inhibitor, we subcutaneously injected 5 × 10^6^ cells into the mice’s contralateral hind legs (FLO-1 in right hind leg and SKGT4 in left hind leg or OE33 in right hind leg and OE33R in left hind leg). 21 days after treatment initiation mice were euthanized and tumor tissues were harvested. Tumor volume was calculated as (W^2^ × L)/2, where W is the smallest diameter of tumor and L is the largest diameter of tumor. Ratio of tumor volume to baseline was calculated for further analysis. The patient derived xenografts (PDXs) were established from a 3-cm subcutaneous metastatic mass from a 45-year-old patient with stage IV esophageal adenocarcinoma [43]. Signs of toxicity including rapid breathing rate, abdominal distension, hunched posture, anorexia, moribund signs were monitored. Gross inspection and microscopic review of Hematoxylin and Eosin (H&E) stained sections from liver, heart, and kidney of the xenografts were reviewed as part of toxicity assessment.

### Histopathology and immunohistochemistry analysis of xenografts

Percentage of tumor cell necrosis and/or stromal fibrosis was assessed in formalin-fixed, paraffin-embedded tumor section stained with H&E. Axl immunohistochemistry was performed on formalin fixed paraffin embedded tissue samples by Axl rabbit monoclonal antibody (Cell Signaling Technology # 8661S) with 15 minutes incubation at room temperature. Endogenous peroxidase activity was blocked by 3% hydrogen peroxidase. The immunoreactive protein was visualized with the Ventana DAB detection system (Dako, Carpenteria, CA). The tumor cells with Axl membranous staining intensity scores of 0, 1, 2, or 3 in 10 fields were counted at 200x magnification. H-score was calculated as sum of the percentage of tumor cells with 0 intensity × 0 + the percentage of tumor cells with staining intensity 1 × 1 + the percentage of tumor cells with staining intensity 2 × 2 + the percentage of staining intensity 3 × 3.

### Stable overexpression of Axl in esophageal adenocarcinoma cell lines

Negative control lentivirus (# LVP-Null-RP) and lentiviral particles with a blasticidin-RFP marker expressing the human gene Axl (# LVP124) were purchased from Amsbio. For transduction with lentivirus, cells were infected with 2× diluted virus media containing 6 μg/ml polybrene for 16 hours. RFP positive cells stably overexpressing Axl were selected by flow cytometry twice. The expression of Axl was confirmed by Western blotting. Cells with negative control were named as FLO-1 RFP and SKGT4 RFP cells while cells stably overexpressing Axl were called FLO-1 Axl and SKGT4 Axl cells.

### Statistical analysis

Each *in vitro* experiment was repeated at least 3 times. For each assay, the one-sided Student *t*-test is used to assess differences between groups. Data is presented as mean ± standard error, and *p* value < 0.05 is considered significant.

## SUPPLEMENTARY MATERIALS





## Abbreviations

CDK9: cyclin dependent kinase 9
CSA: Clonogenic survival assay
GE: gastro-esophageal
H&E: hematoxylin and eosin
i.p.: intraperitoneal
PDX: patient-derived xenografts
RPPA: reverse phase protein array
