# Supplementary material for: Targeting cyclin-dependent kinase 9 by a novel inhibitor enhances radiosensitization and identifies Axl as a novel downstream target in esophageal adenocarcinoma
Source: Oncotarget. 2019 Jul 23;10(45):4703–18. doi: 10.18632/oncotarget.27095 (PMC6659793; doi:10.18632/oncotarget.27095)
Supplement: Supplementary file 1 [file oncotarget-10-4703-s001.pdf]

Targeting cyclin-dependent kinase 9 by a novel inhibitor enhances radiosensitization and identifies Axl as a novel downstream target in esophageal adenocarcinoma

SUPPLEMENTARY MATERIALS

METHODS

Cell lines, cell culture, and CDK9 inhibitors

OE33, FLO-1, and SKGT4 EAC cells were purchased from Sigma-Aldrich (MO). OE33 cells were maintained in RPMI medium containing 2 mM of L-glutamine, 10% fetal bovine serum (FBS), 100 units/ml penicillin, and 100 µg/ml streptomycin. FLO-1 and SKGT4 cells were maintained in Dulbecco's modified Eagle's medium (DMEM) containing 10% FBS, 100 units/ml penicillin, and 100 µg/ml streptomycin. OE33R cells were provided by The Center for Radiation Oncology Research at UT MD Anderson Cancer Center and maintained in RPMI or DMEM. Cells were maintained in a 5% CO_2 atmosphere at 37° C and passaged at 80% confluence. All cell lines were authenticated by MD Anderson's Characterized Cell Line Core Facility. BAY1143572 (#A-1514) was purchased from Active Biochem (NJ).

Cell apoptosis assay, and cell cycle analysis

For apoptosis and cell cycle assays, EAC cells were harvested after treatment with CDK9 inhibitors alone for 48 hours, treatment with radiation alone after 44 hours of incubation with vehicle only, or treatment with BAY1143752 plus radiation. The cells were then washed with cold PBS, resuspended in a solution containing 5 µl of recombinant annexin V-FITC (BD Biosciences, CA) and 5 µg/ml of propidium iodide, and incubated for 15 minutes. Apoptosis and cell cycle were then analyzed by flow cytometry at MD Anderson's Flow Cytometry and Cellular Imaging Facility. Cells stained with propidium iodide alone were considered necrotic, whereas cells stained with annexin V alone were considered apoptotic [1, 2]. The assays were performed in triplicates.

Quantitative reverse transcription polymerase chain reaction

For quantitative reverse transcription polymerase chain reaction (qRT-PCR), total RNA (0.5 µg) isolated from vehicle-treated (control) and BAY1143572 treated cells was reverse-transcribed to cDNA using SuperScript II Reverse Transcriptase (Invitrogen, CA). qPCR was performed using the QuantiFast SYBR Green PCR kit (Qiagen CA). Axl PCR primers were designed using the primer3 program. The ΔCt value of the controls was subtracted from the ΔCt value of each sample normalized to GAPDH to obtain a $\Delta\Delta Ct$ value. The gene expression level relative to the controls was expressed as $2^{-\Delta\Delta Ct}$.

shRNA for genetic downregulation of CDK9 in EAC cell lines

To produce lentivirus that expresses shCDK9, we cotransfected pLKO-shCDK9 (Sigma-Aldrich, St. Louis, MO), or control vectors with their packaging and envelope plasmids into 293FT cells using lipofectamine 2000 reagent according to the manufacturer's instructions (Invitrogen, Carlsbad, CA). Forty eight hours later, viral supernatant was collected after centrifugation at 3000 rpm for 15min. For transduction with lentivirus, cells were infected with 2x diluted virus media containing 6 µg/ml of polybrene for 16 hours. Cells with stably down-regulated CDK9 expression by shCDK9 were selected by incubation in a medium containing purimycin for at least 2 weeks. The expression of target proteins was confirmed by western blot.

Immunoblot analysis

Proteins from cell and tissue lysates were separated using 8% or 10% sodium dodecyl sulfate-polyacrylamide gel electrophoresis. Target protein levels were measured by immunoblotting with rabbit monoclonal antibodies against CDK9 (Cell Signaling Technology# 2316S), Axl (Cell Signaling Technology# 8661S) and Akt (Cell Signaling Technology# 4691S). Bands were visualized by enhanced chemiluminescence (GE Healthcare Life Sciences, PA).

SUPPLEMENTARY METHODS REFERENCES

1. Nicoletti I, Migliorati G, Pagliacci MC, Grignani F, Riccardi C. A rapid and simple method for measuring thymocyte

apoptosis by propidium iodide staining and flow cytometry. Journal of Immunological Methods. 1991; 139:271–9. https://doi.org/10.1016/0022-1759(91)90198-O.

2. Riccardi C, Nicoletti I. Analysis of apoptosis by propidium iodide staining and flow cytometry. Nat Protocols. 2006; 1: 1458–61. https://doi.org/10.1038/nprot.2006.238. [PubMed]

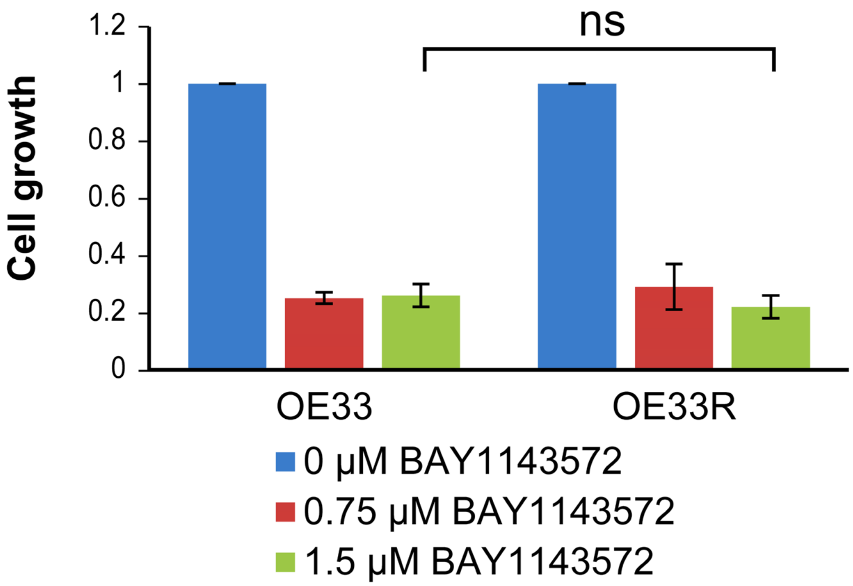

Supplementary Figure 1: CDK9 inhibitors induce apoptosis and sensitize radiation resistant OE33R cells. Cell viability in response to treatment with CDK9 inhibitors in OE33 radiation sensitive and OE33R radiation resistant cells, demonstrated that EAC *in vitro* model of radiation resistant OE33 cells showed similar range of sensitivity as radio naïve OE33 cells when treated with BAY1143572 at the indicated doses for 48 hours and then assessed for cell proliferation. BAY1143572 inhibited proliferation. NS = Not Significant. Data are derived from three independent experiments conducted in triplicate (error bars indicate SEM). Significant differences are indicated (*T/C, *p*-value ≤ 0.05).

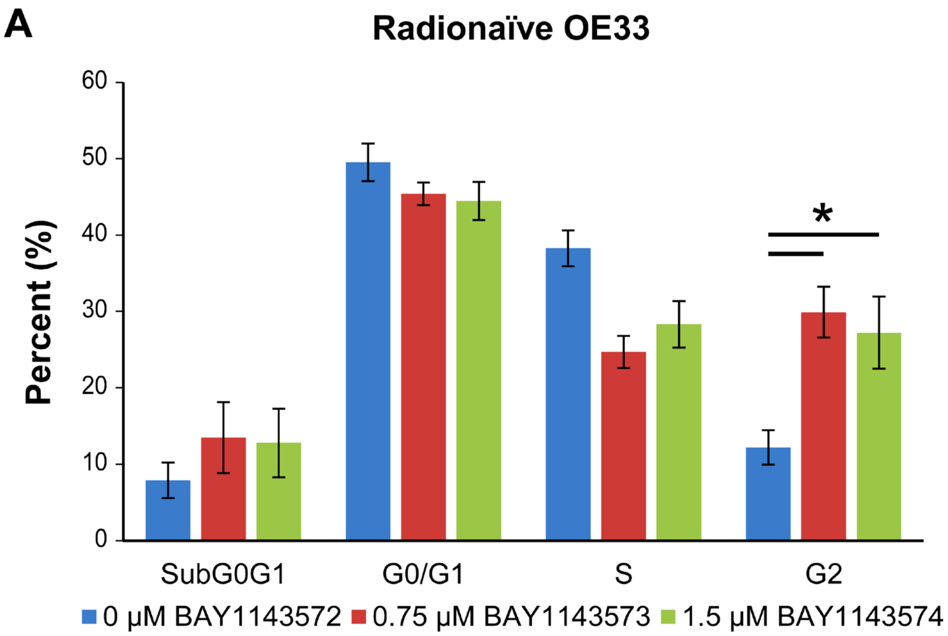

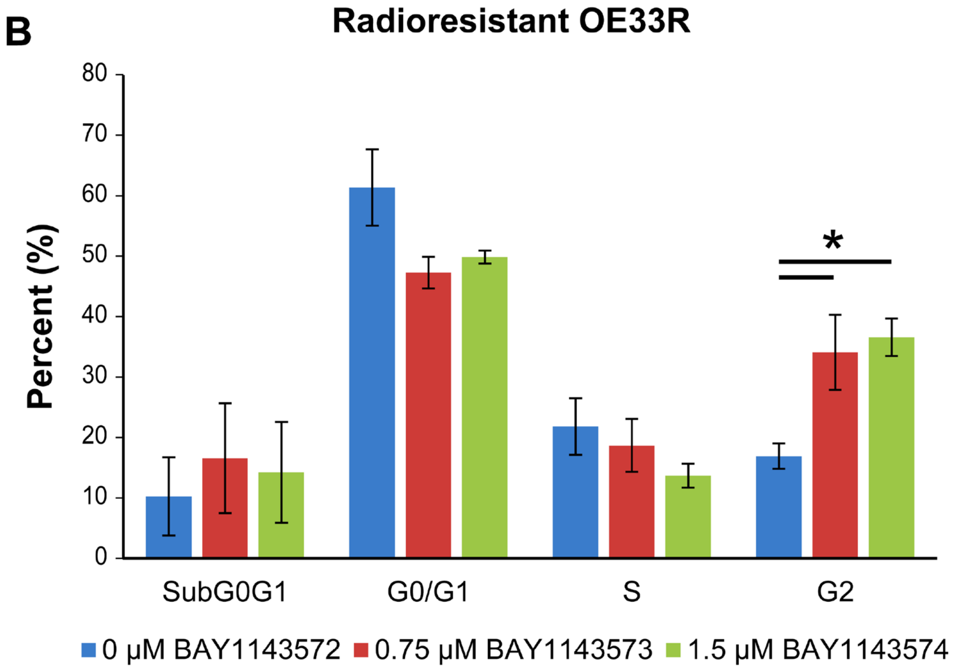

Supplementary Figure 2: CDK9 inhibitors arrest radiation resistant OE33R cells in G2/M phase. Flow cytometry based cell cycle analysis demonstrated that treatment with BAY1143572 for 48 hours caused G2/M arrest in OE33R cells (**B**) which is similar to that of radiation naïve EAC cells (**A**). Data are derived from three independent experiments conducted in triplicate (error bars indicate SEM). Significant differences are indicated (*T/C, *p*-value ≤ 0.05).

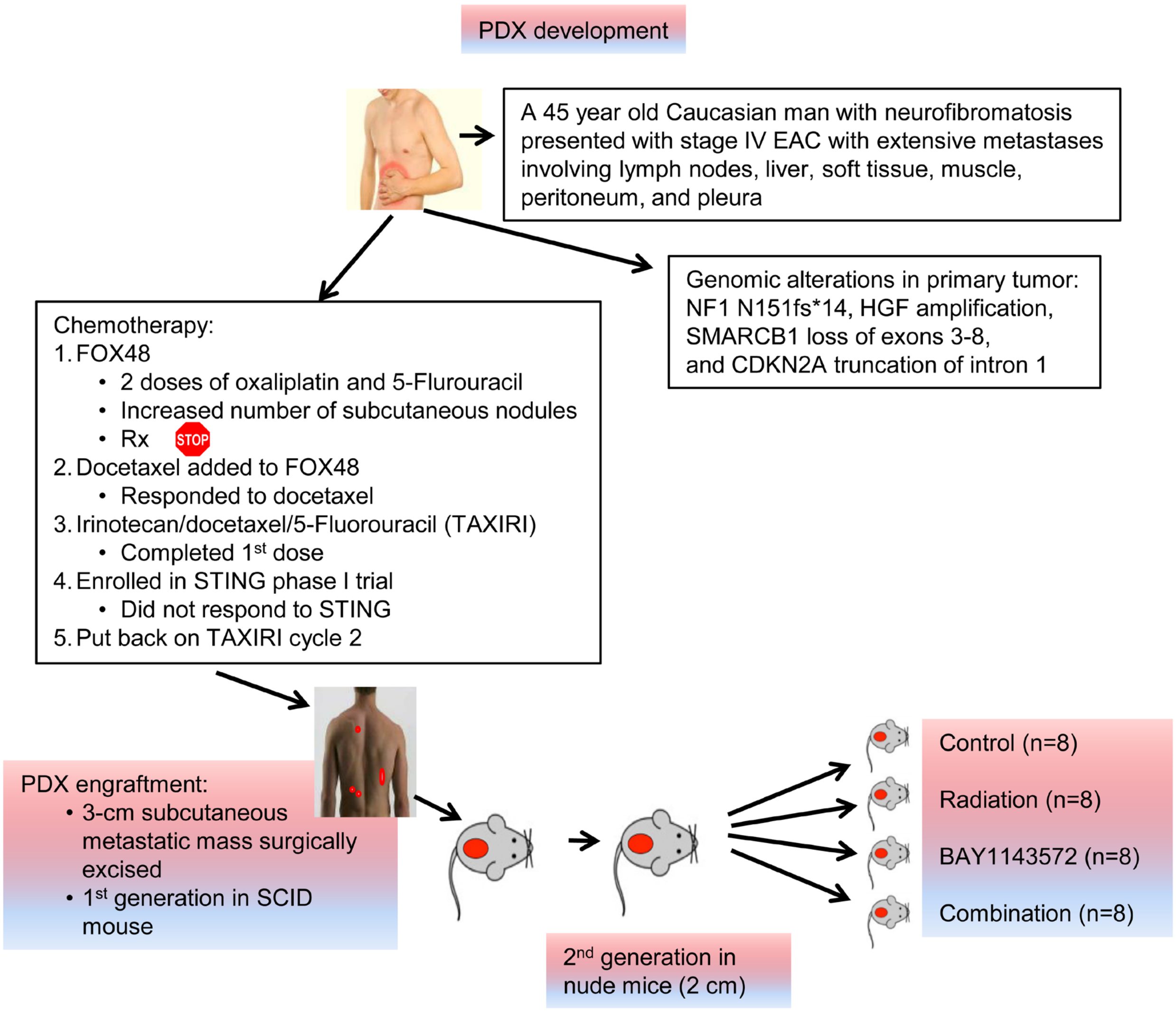

Supplementary Figure 3. Flowchart illustrating the timeline of different treatment regimen for a patient with stage IV EAC and generation of highly refractory EAC PDX. STING (Study of Immunotherapy In Newly Diagnosed Glioblastoma) trial is an immunotherapy based trial for patients with advanced metastatic solid tumors.

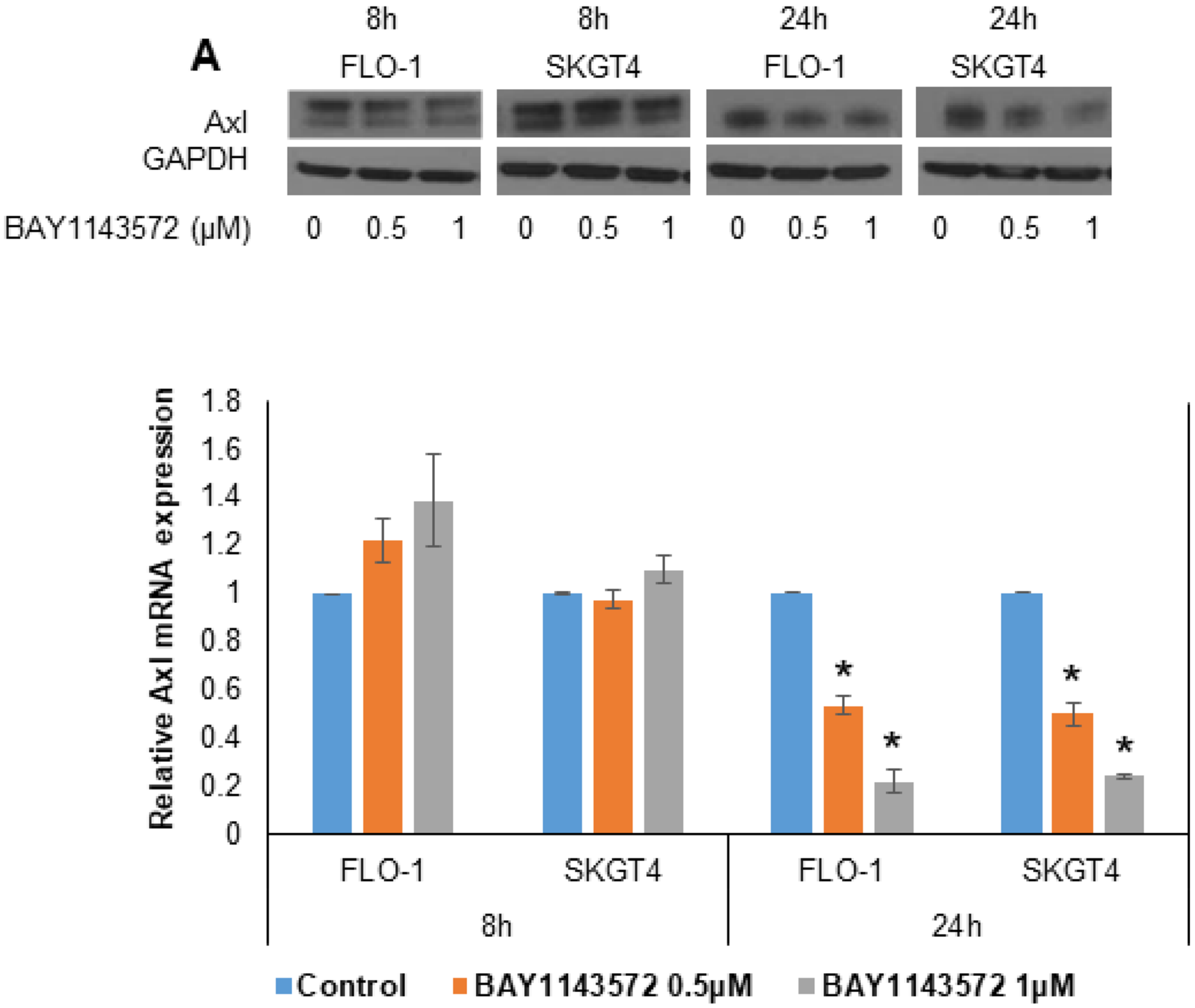

Supplementary Figure 4: Time dependent effect of CDK9 inhibitors on Axl protein and mRNA at 8 and 24 hours. (**A**) Western blot was performed on FLO-1, and SKGT4 cells treated with or without CDK9 inhibitor (BAY, BAY1143572) and checked for Axl protein expression at 8 hour (4 h) and 24 hours (24 h) post treatment. GAPDH was used as loading control. (**B**) qRT-PCR for Axl mRNA in FLO-1 and SKGT4 cells treated by BAY1143572 for 8 h and 24 h. Parental cells treated with DMSO were used as controls in each case. Axl mRNA was normalized to GAPDH mRNA expression in this analysis. Combination groups is not synergistic to individual treatments. The *p*-values were calculated using the Student *t* test; *T/C, *p*-value ≤ 0.05.

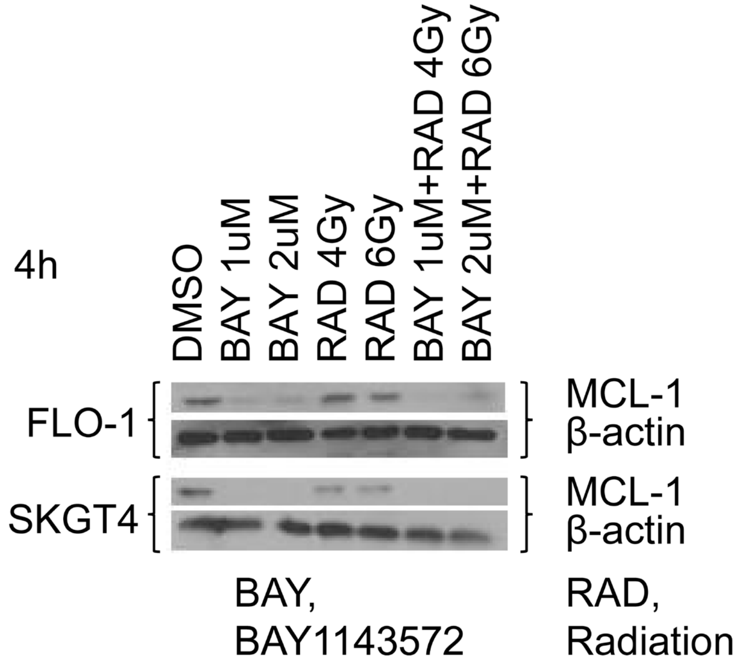

Supplementary Figure 5: Effect of CDK9 inhibitors on MCL-1, target of CDK9 at 4 hours. Western blot was performed on FLO-1, and SKGT4 cells treated with or without CDK9 inhibitors (BAY, BAY1143572) and checked for MCL-1 protein expression at 4 hour (4 h) post treatment. β-actin was used as loading control.

Supplementary Table 1: Protein biomarkers altered by BAY1143572 in EAC cells by RPPA-based proteomics. The protein list of AUC ≥ 0.5 in RPPAs. Differential proteins with negative values are downregulated while positive values are upregulated compared to control. See Supplementary_Table_1
