# Supplementary material for: Targeting cyclin-dependent kinase 9 by a novel inhibitor enhances radiosensitization and identifies Axl as a novel downstream target in esophageal adenocarcinoma
Source: Oncotarget. 2019 Jul 23;10(45):4703–18. doi: 10.18632/oncotarget.27095 (PMC6659793; doi:10.18632/oncotarget.27095)
Supplement: Supplementary file 2 [file oncotarget-10-4703-s002.docx]

**Supplementary Table 1. Protein biomarkers altered by BAY1143572 in EAC cells by RPPA-based proteomics.** The protein list of AUC≥0.5 in RPPAs. Differential proteins with negative values are downregulated while positive values are upregulated compared to control.

|  | **Cell Line** | | |
| --- | --- | --- | --- |
| **Altered proteins** | **FLO-1** | **SKGT4** | **OE33** |
| ADAR1 | -0.0524 | 0.18784 | 0.12526 |
| AKT | 0.56276 | 0.39735 | 0.08698 |
| AKT_pT308 | 0.00523 | -0.1988 | 0.10877 |
| Bcl-2 | 0.30866 | 0.31146 | 0.03714 |
| CD29 | 0 | 0.26911 | 0.10634 |
| CD31 | 0 | 0.25124 | 0.14355 |
| Cyclin-B1 | 0.12422 | 0.27708 | -0.6144 |
| ERCC1 | -0.0662 | 0.10325 | 0.15004 |
| FASN | 0.42481 | 0.17746 | -0.1493 |
| FoxM1 | 0.11023 | 0.15977 | 0.15832 |
| Granzyme B | 0.26149 | 0.18732 | 0.13927 |
| Gys | 0.23182 | 0.10117 | -0.1482 |
| MERIT40_pS29 | -0.038 | 0.2013 | 0.11644 |
| NDUFB4 | 0.22633 | 0.0026 | 0.22865 |
| N-Ras | 0.10709 | 0.21496 | 0.14427 |
| p44-42-MAPK | 0.25839 | 0.23629 | -0.1488 |
| PAICS | 0.27489 | 0.14064 | -0.048 |
| Paxillin | 0.17389 | 0.40694 | -0.0698 |
| PKA-a | 0.17827 | 0.28971 | -0.3935 |
| Porin | -0.2051 | 0.39469 | 0.19027 |
| S6 | 0.28355 | 0.27766 | 0.09361 |
| Smad1 | 0.23569 | -0.0837 | 0.13554 |
| Syk | 0.05912 | 0.2476 | 0.22242 |
| Transglutaminase | 0.07976 | 0.1275 | 0.1672 |
| TUFM | -0.1849 | 0.30373 | 0.32269 |
| UGT1A | 0.2094 | 0.28332 | 0.24893 |
| 4E-BP1_pS65 | 0.03997 | -0.3173 | -0.6993 |
| ACC_pS79 | 0.30159 | -0.4855 | -0.6526 |
| Akt_pS473 | 0.13364 | -0.3536 | -0.1962 |
| Aurora-B | -0.0453 | -0.3441 | -0.1678 |
| Axl | -0.7921 | 0.26504 | -0.4489 |
| Bad_pS112 | -0.0013 | -0.1972 | -0.3101 |
| Bax | 0.02485 | -0.2827 | -0.293 |
| b-Catenin | -0.7317 | 0.07553 | -0.1881 |
| Bim | -0.3065 | -0.2196 | 0.16798 |
| B-Raf | -0.0468 | -0.1214 | -0.2325 |
| B-Raf_pS445 | 0.27587 | -0.2 | -1.0316 |
| Caveolin-1 | -0.355 | 0.14379 | -0.2736 |
| cdc25C | 0.2374 | -0.3784 | -0.4968 |
| Chk1 | 0.16823 | -0.2232 | -0.174 |
| COX-IV | -0.4392 | -0.166 | -0.1412 |
| C-Raf_pS338 | -0.0471 | -0.1469 | -0.4112 |
| Cyclin-D3 | -0.0356 | -0.3508 | -0.3411 |
| DUSP4 | -0.2763 | -1.1818 | -0.8502 |
| E-Cadherin | -0.5403 | -0.3712 | -0.1975 |
| eEF2K | 0.3049 | -0.4043 | -0.3468 |
| EGFR | -0.1398 | -0.0564 | -0.3286 |
| FAK_pY397 | -0.149 | -0.2212 | -0.1224 |
| Gab2 | 0.23791 | -0.3095 | -0.2132 |
| HER2 | -0.3824 | -0.4281 | -0.2632 |
| HES1 | -0.222 | -0.3935 | -0.4488 |
| Hexokinase II | -0.1917 | -0.0757 | -0.3048 |
| IGFBP2 | -0.4879 | -2.6481 | 0.15056 |
| IRS1 | -0.0006 | -0.2609 | -0.146 |
| Jagged1 | -0.1252 | -0.3316 | -1.0524 |
| LRP6_pS1490 | -0.2878 | -0.2298 | -0.6566 |
| Lck | -0.3808 | -1.1324 | 0.06218 |
| MCL-1 | -0.1752 | -0.7067 | -0.164 |
| MCT4 | -0.4974 | 0.03803 | -0.2395 |
| MDM2_pS166 | -0.1019 | -0.1468 | 0.12091 |
| Mnk1 | 0.18246 | -0.6445 | -0.2685 |
| NDRG1_pT346 | 0.31515 | -0.2238 | -1.1687 |
| p16INK4a | 0.66061 | -0.1157 | -0.5123 |
| p38-MAPK | 0.22303 | -0.3152 | -0.1777 |
| PAI-1 | 0.0043 | 0.32401 | -0.1469 |
| PAK1 | 0.07799 | -0.1887 | -0.2303 |
| PARP1 | -0.2948 | 0.31646 | -0.1702 |
| PDGFR-b | -0.3049 | -0.2895 | 0.12417 |
| PDK1_pS241 | 0.1112 | -0.217 | -0.2464 |
| PKC-b-II_pS660 | -0.1137 | -0.1472 | -0.1429 |
| PMS2 | -0.1114 | -0.1535 | -0.2867 |
| Rad51 | 0.22256 | -0.4476 | -0.1683 |
| Rb_pS807_S811 | 0.16258 | -0.4118 | -0.4388 |
| S6_pS235_S236 | 0.22575 | -0.5953 | -0.1162 |
| S6_pS240_S244 | 0.09749 | -0.5438 | -0.1309 |
| SCD | -1.3032 | -0.0845 | -0.3015 |
| Stat3_pY705 | -0.1763 | -0.1067 | -0.1632 |
| TAZ | 0.41443 | -0.1212 | -0.2411 |
| Tyro3 | -0.0847 | -0.1219 | -0.2011 |
| YB1_pS102 | -0.0214 | -0.1346 | -0.1685 |
